# Factors Affecting Risk Perception of Electromagnetic Waves From 5G Network Base Stations

**DOI:** 10.1002/bem.22290

**Published:** 2020-08-31

**Authors:** Tae Hwan Koh, Jae Wook Choi, Myungsoon Seo, Hyung‐Do Choi, KyungHee Kim

**Affiliations:** ^1^ Department of Occupational and Environmental Medicine Korea University Ansan Hospital Ansan Republic of Korea; ^2^ Korea University College of Medicine and School of Medicine Seoul Republic of Korea; ^3^ Department of Public Health Korea University Seoul Republic of Korea; ^4^ EM Environment Research Team Electronics and Telecommunications Research Institute Daejeon Republic of Korea; ^5^ Institute for Occupational and Environmental Health Korea University Seoul Republic of Korea

**Keywords:** 5G network, base stations, electromagnetic waves, risk perception, Korea

## Abstract

The coverage of the fifth‐generation network has increased steadily since the network was introduced in 2019. However, public protests around the globe against the construction of 5G network base stations have continued to occur for fear that electromagnetic (EM) waves emitted from the stations would cause adverse health effects. To identify factors that have contributed to such increased risk perception, we conducted a cross‐sectional study using data obtained from a survey that assessed Korean adults’ risk perception of EM wave‐related objects. We found that female gender, high level of perceived exposure to EM waves, evaluation of public policies as ineffective, and high level of objective knowledge on EM waves were associated with increased risk perception. Furthermore, we found that higher ratings on a few risk characteristics such as “personal knowledge,” “seriousness of the risk to future generations,” “dreadfulness,” and “severity of consequences” were also associated with increased risk perception as well. Bioelectromagnetics. © 2020 The Authors. *Bioelectromagnetics* published by Wiley Periodicals LLC on behalf of Bioelectromagnetics Society

## INTRODUCTION

The recent development of the fifth‐generation (5G) network has led to widespread expectations that it would revolutionize mobile communications. As such, demand for the 5G network has been high; Korean mobile carriers began distributing the service in April 2019 [France‐Presse, 2019], and in less than 3 months, more than a million users have joined the network [Chamberlain, 2019]. Since then, other countries have also witnessed steady increases in the number of subscribers, leading experts to project that 5G network would cover 55–65% of the world population by 2025 [Reichert, 2019].

In contrast to its apparent popularity, however, the new technology has not been welcomed by everyone. Many members of the public have perceived that electromagnetic (EM) waves from 5G network base stations are potential health risks, which has been shown by the protests in Korea that have delayed construction of base stations [Jun, 2019], and anti‐5G demonstrations that filled the streets in the United Kingdom [Hern, 2019] and Switzerland [Jones, 2020]. Authorities such as the World Health Organization [2020] made statements that EM waves from 5G network base stations would not cause substantial adverse health effects as their levels were well within the exposure limits; however, the public risk perception has not diminished much.

What are the factors that have contributed to such public risk perception? Risk perception research has answered such a question by analyzing people's ability to make health‐related choices [Fischhoff et al., 1993]; in fact, studies on objects that are qualitatively similar to EM waves from 5G network base stations have been published. Tseng et al. [2013] found that psychopathology and sensitivity to the electromagnetic field (EMF) were associated with increased risk perception on different sources of EMF. And Kowall et al. [2012] reported that female gender, a higher level of education, visibility of mobile phone base stations, and a higher level of negative emotions were associated with increased risk perception of mobile phone base stations. Furthermore, Kim et al. [2014] found that higher ratings on a few risk characteristics such as “personal knowledge,” “outrage,” and “seriousness of the risk to future generations” were associated with increased risk perception of mobile phones.

However, perhaps owing to the newness of the 5G network's development, no study has been published on risk perception of EM waves from 5G network base stations. Therefore, we conducted a cross‐sectional study using the data from a survey that assessed Korean adults’ risk perception of EM wave‐related objects. Our aims were to assess the degree of risk perception of EM waves from 5G network base stations and identify variables that significantly influenced the risk perception.

## MATERIALS AND METHODS

### Study Subjects

From 1.1 million Koreans who were registered in the access panel of Truis (a market research firm in Seoul, Korea), 207,809 adults over the age of 19 were selected by stratified random sampling (by gender, age group, and residential area) and contacted by e‐mail and KakaoTalk (a messaging application used widely in Korea) to participate in our survey. The response rate was 2.7%, as 5,677 responded to the survey. After taking quality control measures including exclusion of those who left the survey unfinished, data on 3,393 Korean adults were available for analysis. For the purpose of quality assurance, study subject selection, and coding of the data were conducted by Truis.

### Survey

The survey was designed to measure the degree of Korean adults’ risk perception of EM wave‐related objects, and it consisted of eight questionnaires, which were as follows: SQ (screening questions), DQ (demographic questions), section A (EM wave‐related sources), section B (objective knowledge), section C (risk perception and risk characteristics), section D (protective measures), section E (symptoms associated with use of EM wave‐related sources), and section F (media exposure and public policies). The study subjects completed the survey by accessing a web‐based program set up by Truis in 2019.

### Variables of Interest

Questions from the survey were screened to identify variables that would be relevant for our analysis. Demographic variables including gender, age group, marital status, level of education, monthly household income, smoking status, drinking status, whether study subjects lived with seniors with chronic disease, whether they lived with juniors younger than middle school age, and whether they lived with females (or were the ones) who were either pregnant or planning to become pregnant were selected.

EM wave exposures and health‐related variables including whether study subjects used a mobile phone, whether they placed a mobile phone charger nearby while asleep, level of perceived exposures to EM waves, evaluation of public policies that provide protection from EM wave exposures, level of objective knowledge on EM waves, and self‐reported health were selected. Of note, the level of objective knowledge on EM waves was assessed by section B from our survey, and its details are provided in Supplementary Table 1.

Risk characteristics [Slovic, 1987] assessed by section C of our survey, which included “personal knowledge,” “controllability,” “seriousness of the risk to future generations,” “dreadfulness,” “severity of consequences,” “risk known to science,” “immediacy of the effect of risk,” and “familiarity” were also selected. Details of questions and 10‐point scales that were used to evaluate EM waves from 5G network base stations in regards to the risk characteristics are provided in Supplementary Table 2.

Lastly, risk perception score was included; the score was rated on a 10‐point scale where 1 signified the least severe risk and 10 the most severe risk. In our survey, the study subjects rated risk perception score on a total of 19 objects. Thirteen objects were EM wave‐related; they were EM waves from 5G base stations, EM waves from mobile phones, EM waves from microwaves, EM waves from air fryers, EM waves from hair dryers, EM waves from massage chairs, EM waves from electronic foot baths, EM waves from low‐frequency therapy devices, EM waves from electric shavers, EM waves from radars, EM waves from transmission lines, EM waves from Bluetooth devices, and EM waves from electric heaters. On the contrary, six were not EM wave‐related; they were household chemical products, climate change, micro‐dust, drinking water pollution, electronic cigarettes, and cigarette smoking.

### Statistical Analysis

Descriptive statistical analysis on demographic variables, EM wave exposure and health‐related variables, risk characteristics, and risk perception score was carried out. *T* test was conducted to check for the presence of significant differences in risk perception scores of EM wave‐related objects between the two groups classified based on the level of objective knowledge.

Multiple linear regression was conducted to identify factors that significantly influenced risk perception of EM waves from 5G network base stations. Risk perception score of EM waves from 5G network base stations was chosen as the dependent variable; demographic variables, EM wave exposure and health‐related variables, and risk characteristics were used as covariates. Slopes and 95% confidence intervals (CIs) were calculated to quantify the magnitude of impact that each covariate exerted on the risk perception score of EM waves from 5G network base stations. All statistical analyses were performed using SPSS ver. 22 (IBM, Armonk, NY).

### Ethics Statement

This study was conducted after obtaining approval from the Institutional Review Board (IRB) of Korea University (KUIRB‐2019‐0240‐01).

## RESULTS

### Demographic Characteristics

All of the study subjects were Koreans over the ages of 19; 51.9% (*n *= 1,760) were male, and 48.1% (*n* = 1,633) female. For age group, 22.5% (*n* = 762) were between 20 and 29 years of age, 23.8% (*n* = 807) between 30 and 39, 27.9% (*n* = 945) between 40 and 49, and 25.9% (*n* = 879) between 50 and 59. For marital status, 43.5% (*n* = 1,677) were single, 53.6% (*n* = 1,818) married, and 2.8% (*n* = 94) either divorced or widowed. For level of education, 17.5% (*n* = 593) were high school graduates or less, 52.1% (*n* = 1,769) either college students or graduates, and 30.4% (*n* = 1,031) more than college graduates. The distribution of monthly household income was unimodal with 46.9% (*n* = 1,593) earning between 3,000 and 6,000 dollars. For smoking status, 55.8% (*n* = 1,894) were never‐smokers, 18.4% (*n* = 624) past smokers, and 25.8% (*n* = 875) current smokers. For drinking status, 17.2% (*n* = 584) were never‐drinkers, 25.8% (*n* = 874) past drinkers, and 57.0% (*n* = 1,935) current drinkers. For living with vulnerable individuals, 17.5% (*n *= 593) lived with seniors diagnosed with chronic disorders, 23.5% (*n* = 799) with juniors younger than middle school age, and 7.2% (*n* = 243) with females (or were the ones) who were pregnant or planning to become pregnant (Table 1).

**Table 1:** Demographic Characteristics (*N* = 3,393)

Variables	*N*	%
Gender		
Male	1,760	51.9
Female	1,633	48.1
Age group		
20–29	762	22.5
30–39	807	23.8
40–49	945	27.9
50–59	879	25.9
Marital status		
Single	1,677	43.5
Married	1,818	53.6
Divorced or widowed	94	2.8
Others	4	0.1
Level of education		
A high school graduate or less	593	17.5
A college student or graduate	1,769	52.1
More than a college graduate	1,031	30.4
Monthly household income		
<3,000 dollars	965	28.4
<6,000 dollars	1,593	46.9
≥6,000 dollars	835	24.6
Smoking status		
Never‐smoker	1,894	55.8
Past smoker	624	18.4
Current smoker	875	25.8
Drinking status		
Never‐drinker	584	17.2
Past drinker	874	25.8
Current drinker	1,935	57.0
Lives with seniors		
Yes	593	17.5
No	2,800	82.5
Lives with juniors		
Yes	799	23.5
No	2,594	76.5
Lives with pregnant females		
Yes	243	7.2
No	3,150	92.8

### EM Wave Exposures and Health‐Related Characteristics

Most of the study subjects (99.9%; *n* = 3,388) used a mobile phone, and 66.4% (*n* = 2,248) placed a mobile phone charger nearby while asleep. About eighty percent (77.9%; *n* = 2,643) perceived that they were exposed to a high level of EM waves. In evaluating public policies that provide protection from EM wave exposures, 19.9% (*n* = 675) considered them effective, 54.3% (*n* = 1,841) average, and 26.8% (*n* = 877) ineffective. The mean score on section B, which tested the level of objective knowledge on EM waves, was 4.6 problems correct out of 13 problems; 49.5% (*n* = 1,679) scored better than the mean, and 50.5% (*n* = 1,714) lower than the mean. For self‐reported health, 45.2% (*n* = 1,535) considered themselves to be healthy, and 54.8% (*n* = 1,858) unhealthy (Table 2).

**Table 2:** Electromagnetic Wave Exposures and Health‐Related Characteristics (*N* = 3,393)

Variables	*N*	%
Uses a mobile phone		
Yes	3,388	99.9
No	5	0.1
Places a mobile phone charger nearby while asleep		
Yes	2,248	66.4
No	1,140	33.6
Level of perceived exposures to EM waves		
High	2,643	77.9
Low	750	22.1
Evaluation of public policies that provide protection from EM wave exposures		
Effective	675	19.9
Average	1,841	54.3
Ineffective	877	26.8
Level of objective knowledge on EM waves		
High^a^	1,679	49.5
Low^b^	1,714	50.5
Self‐reported health		
Healthy	1,535	45.2
Unhealthy	1,858	54.8

^a^High: study subjects who answered 5–13 questions correctly (above the mean score 4.6/13).

^b^Low: study subjects who answered 0–4 questions correctly (below the mean score of 4.6/13).

### Risk Characteristics of EM Waves From 5G Network Base Stations

The mean score on “personal knowledge” was 5.08 on a 10‐point scale (1: not knowledgeable and 10: very knowledgeable). The mean score on “controllability” was 4.26 (1: uncontrollable and 10: very controllable). The mean score on “seriousness of the risk to future generations” was 6.33 (1: not serious and 10: very serious). The mean score on “dreadfulness” was 5.77 (1: not dreadful and 10: very dreadful). The mean score on “severity of consequences” was 5.91 (1: not severe and 10: very severe). The mean score on “risk known to science” was 4.84 (1: not known to science and 10: well known to science). The mean score on “immediacy of effect of risk” was 4.60 (1: happens slowly and 10: happens immediately). The mean score on “familiarity” was 4.64 (1: unfamiliar and 10: very familiar) (Supplementary Table 2).

### Risk Perception Score

**Figure 1:**
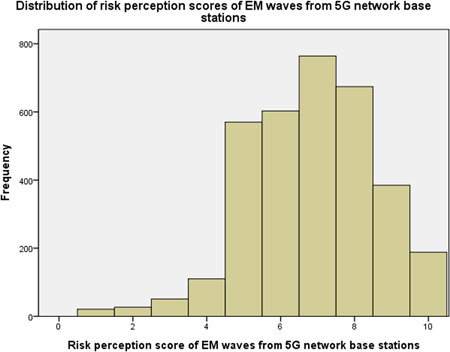
Distribution of risk perception scores of EM waves from 5G network base stations (*N *= 3,393). EM, electromagnetic.

The distribution of risk perception scores of EM waves from 5G network base stations was unimodal and approximately symmetric (Fig. 1); the mean risk perception score was 6.84 on a 10‐point scale, which was the fifth‐highest among 13 EM wave‐related objects. Only EM waves from transmission lines (7.94), EM waves from radars (7.21), EM waves from microwaves (7.01), and EM waves from mobile phones (6.85) were perceived as more severe risks. When all the nineteen objects were taken into account, EM waves from 5G network base stations exhibited the 10th highest mean risk perception score. Risk perception scores of EM waves from mobile phones showed the highest Pearson correlation coefficient (0.545) with those of EM waves from 5G network base stations, while risk perception scores of drinking water pollution showed the lowest Pearson correlation coefficient (0.240) (Table 3).

**Table 3:** Risk Perception Scores of Various Objects (*N* = 3,393)

Object	Mean risk perception score (SD)	Median risk perception score (25th, 75th percentile)	Correlation coefficient with risk perception scores of EM waves from 5G network base stations^a^	Rank total/EM waves‐related only
EM waves from 5G network base stations	6.84 (1.71)	7 (6, 8)	1	10th/5th
EM waves from mobile phones	6.85 (1.63)	7 (6, 8)	0.545	9th/4th
EM waves from microwaves	7.01 (1.72)	7 (6, 8)	0.485	8th/3rd
EM waves from air fryers	6.29 (1.75)	6 (5, 8)	0.472	13th/7th
EM waves from hair dryers	6.13 (1.73)	6 (5, 7)	0.477	16th/10th
EM waves from massage chairs	6.23 (1.68)	6 (5, 7)	0.493	14th/8th
EM waves from electronic foot baths	5.83 (1.69)	6 (5, 7)	0.457	18th/12th
EM waves from low‐frequency therapy devices	5.97 (1.76)	6 (5, 7)	0.457	17th/11th
EM waves from electric shavers	5.60 (1.79)	5 (5, 7)	0.434	19th/13th
EM waves from radars	7.21 (1.83)	7 (6, 9)	0.537	6th/2nd
EM waves from transmission lines	7.94 (1.79)	8 (7, 9)	0.499	4th/1st
EM waves from Bluetooth devices	6.15 (1.71)	6 (5, 7)	0.520	15th/9th
EM waves from electric heaters	6.60 (1.67)	7 (5, 8)	0.495	12th/6th
Household chemical products	6.70 (1.64)	7 (6, 8)	0.443	11th/‐
Climate change	7.17 (1.88)	7 (6, 9)	0.335	7th/‐
Micro‐dust	8.11 (1.70)	8 (7, 10)	0.317	3rd/‐
Drinking water pollution	7.44 (2.01)	8 (6, 9)	0.240	5th/‐
Electronic cigarettes	8.27 (1.74)	9 (7, 10)	0.302	2nd/‐
Cigarette smoking	8.50 (1.74)	9 (8, 10)	0.278	1st/‐

EM, electromagnetic.

^a^Pearson correlation coefficient was calculated to measure the strength of association between two objects’ risk perception scores.

The mean risk perception score of EM waves from 5G network base stations was significantly higher for study subjects with a high level of objective knowledge than those with a low level of objective knowledge. And the same pattern was observed in regards to twelve other EM wave‐related objects (Supplementary Table 3).

### Multiple Linear Regression on Risk Perception Scores of EM Waves From 5G Network Base Stations

Risk perception scores of EM waves from 5G network base stations were higher among female (*β*: 0.188, *P*: 0.001; reference: male) study subjects who perceived that they were exposed to a high level of EM waves (*β*: 0.442, *P*: <0.001; reference: low), those who regarded public policies as ineffective (*β*: 0.124, *P*: 0.035; reference: average), and those with a high level of objective knowledge (*β*: 0.176, *P*: <0.001; reference: low). Higher ratings on risk characteristics such as “personal knowledge” (*β*: 0.112, *P*: <0.001), “seriousness of the risk to future generations” (*β*: 0.243, *P*: <0.001), “dreadfulness” (*β*: 0.066, *P*: <0.001), and “severity of consequences” (*β*: 0.135, *P*: <0.001) were also associated with higher risk perception scores (Table 4).

**Table 4:** Multiple Linear Regression^a,b,*^ of Risk Perception Scores of EM Waves From 5G Network Base Stations (*N* = 3,388)

Variables	Non‐standardized β (SE)/standardized β	95% CIs		*P* value
Gender			
Male	Reference		
Female	0.188 (0.056)/0.055	0.079	0.298	0.001
Age group				
20–29	−0.261 (0.090)/−0.064	−0.436	−0.085	0.004
30–39	−0.025 (0.080)/−0.006	−0.182	0.133	0.758
40–49	0.005 (0.072)/0.001	−0.135	0.146	0.940
50–59	Reference			
Marital status				
Married	−0.022 (0.070)/−0.006	−0.159	0.116	0.758
Others	Reference			
Level of education				
A high school graduate or less	−0.051 (0.075)/−0.011	−0.198	0.095	0.492
A college student or graduate	−0.019 (0.056)/−0.006	−0.128	0.090	0.730
More than a bachelor's degree	Reference			
Monthly household income				
<3,000 dollars	−0.040 (0.062)/−0.011	−0.161	0.081	0.518
<6,000 dollars	Reference	−0.168	0.068	0.407
≥6,000 dollars	−0.050 (0.060)/−0.013			
Smoking status				
Never	Reference			
Past smoker	−0.084 (0.072)/−0.019	−0.224	0.057	0.243
Current smoker	−0.138 (0.067)/−0.035	−0.269	−0.008	0.038
Drinking status				
Never	−0.244 (0.071)/−0.054	−0.382	−0.105	0.001
Past drinker	−0.058 (0.059)/−0.015	−0.174	0.059	0.331
Current drinker	Reference			
Lives with seniors				
Yes	Reference			
No	0.027 (0.065)/0.006	−0.100	0.155	0.673
Lives with juniors				
Yes	0.036 (0.070)/0.009	−0.102	0.173	0.611
No	Reference			
Lives with pregnant females				
Yes	Reference			
No	0.037 (0.097)/0.006	−0.153	0.226	0.704
Places a mobile phone charger nearby while asleep				
Yes	−0.060 (0.052)/−0.017	−0.162	0.041	0.244
No	Reference			
Level of perceived exposures to EM waves				
High	0.442 (0.061)/0.107	0.323	0.562	<0.001
Low	Reference			
Self‐reported health				
Healthy	0.031 (0.050)/0.009	−0.068	0.130	0.538
Unhealthy	Reference			
Evaluation of public policies that provide protection from EM wave exposures				
Effective	0.037 (0.064)/0.009	−0.088	0.162	0.563
Average	Reference	0.009	0.239	0.035
Ineffective	0.124 (0.059)/0.032			
Level of objective knowledge on EM waves				
Low	Reference			
High	0.176 (0.050)/0.052	0.079	0.274	<0.001
Personal knowledge				
Numerical	0.112 (0.013)/0.151	0.086	0.138	<0.001
Controllability				
Numerical	−0.037 (0.012)−0.052	−0.060	−0.014	0.001
Seriousness of risk to the future generations				
Numerical	0.243 (0.015)/0.291	0.213	0.272	<0.001
Dreadfulness				
Numerical	0.066 (0.016)/0.087	0.035	0.097	<0.001
Severity of consequences				
Numerical	0.135 (0.016)/0.171	0.104	0.167	<0.001
Risk known to science				
Numerical	−0.034 (0.014)/−0.047	−0.060	−0.007	0.013
Immediacy of effect of risk				
Numerical	−0.012 (0.013)/−0.016	−0.037	0.014	0.370
Familiarity				
Numerical	−0.002 (0.014)/−0.003	−0.029	0.025	0.887

EM, electromagnetic; SE, standard error.

^a^Covariates were mutually adjusted.

^b^ *R* ^2^ of the model: 0.339.

*P for the *F*‐test: <0.001.

On the contrary, risk perception scores were lower among current smokers (β: −0.138, *P*: 0.0358; reference: never‐smokers), never‐drinkers (*β*: −0.244, *P*: 0.001; reference: current drinkers), and study subjects who were between 20 and 29 years of age (*β*: −0.261, *P*: 0.004; reference: 50–59). Higher ratings on risk characteristics such as “controllability” (*β*: −0.037, *P*: 0.001) and “risk known to science” (*β*: −0.034, *P*: 0.013) were also associated with lower risk perception scores (Table 4).

## DISCUSSION

Risk communication used to depend solely on calculating mortality estimates and releasing the numbers to the public, hoping that such action would reduce concerns; however, even when experts and the public saw the same estimates, they still disagreed on the magnitude of perceived risks [Fischhoff, 1995]. It is now well recognized that such disagreement occurred as members of the public took many factors into consideration, in addition to objective measures of hazards when they perceived risks [Sandman, 1993]. And we aimed to identify such factors that influenced public risk perception of EM waves from 5G network base stations.

Our study found that EM waves from 5G network base stations were perceived as moderate health risks; the magnitude of the perceived risk was similar to that of EM waves from mobile phones, greater than that of household chemical products, but less than that of cigarette smoking. Furthermore, the risk perception of EM waves from 5G network base stations was related the most closely to that of EM waves from mobile phones and the least close to that of drinking water pollution.

Factors associated with increased risk perception of EM waves from 5G network base stations were identified. The risk perception scores were higher among female study subjects who perceived that they were exposed to high levels of EM waves, and those who rated public policies as ineffective. Furthermore, study subjects who gave higher ratings on “personal knowledge,” “dreadfulness,” “seriousness of the risk to future generations,” and “severity of consequences” also exhibited higher risk perception scores. Such results were consistent with those from past risk perception studies. Gustafson [1998] found that females tended to evaluate risks as more severe; in particular, Siegrist et al. [2005] reported that females viewed technological risks as more severe than males. Freudenstein et al. [2015] found that a higher level of perceived exposures to radiofrequency EMF was associated with an increased risk perception of radiofrequency EMF. A lower level of trust in government policies was also associated with an increased risk perception of EMF sources [van Dongen et al., 2013]. Furthermore, Kim et al. [2014] revealed that people who gave higher ratings on “personal knowledge” and “seriousness of the risk to future generations” also exhibited increased risk perception of EMF from mobile phones.

Factors associated with decreased risk perception of EM waves from 5G network base stations were also identified. The risk perception scores were lower among the 20–29 age group, current smokers, and never‐drinkers. Furthermore, study subjects who gave higher ratings on “controllability” and “risk known to science” exhibited lower risk perception scores. Part of the results was supported by past findings. Bonem et al. [2015] found that older adults evaluated health and safety‐related risks as more severe than younger adults; similarly, Morgan et al. [2019] reported that older adults showed more severe risk estimation compared with younger adults. Further, Ho et al. [2008] found that an increased sense of controllability was associated with decreased risk perception.

However, some of our results differed from past findings and our initial expectations. Dosman et al. [2001] found that a longer duration of education was associated with decreased risk perception, but the level of education did not affect the risk perception in our analysis. Furthermore, Lemyre et al. [2006] demonstrated that lower income was associated with increased risk perception; however, monthly household income did not affect the risk perception in our study. Such differences might be due to different risk factors and study populations that were investigated, although a separate study is warranted to formulate a definite conclusion. Furthermore, we had expected that current drinkers would have decreased risk perception of EM waves from 5G network base stations compared to never‐drinkers, as we postulated that those who were already engaged in risky behavior of drinking alcohol would be more tolerant to other potential health risks; but the result was completely the opposite. We also had expected to find that living with vulnerable groups (seniors, juniors, and pregnant females) would be associated with increased risk perception, but no significant association was observed. Furthermore, we had hypothesized that a behavior of placing a mobile phone charger nearby while asleep would be associated with lower risk perception as the behavior is suggestive of indifference to exposure to EM waves; however, no significant relationship was observed as well.

Lastly, we focus our discussion on the association between objective knowledge and risk perception. Objective knowledge can be enhanced effectively by education; hence, it is likely to become a policy target for risk communication efforts in the future. Past studies on the association between objective knowledge and risk perception have shown mixed results. Cousin and Siegrist [2011] showed that when Swiss citizens read a booklet on mobile communications, their objective knowledge improved but at the cost of increasing concerns concomitantly. On the contrary, Claassen et al. [2017] reported that providing the public with information on exposure to EMF improved knowledge and reduced risk perception. In our study, study subjects who scored above the mean on section B considered EM waves from 5G network base stations as more risky than those who scored below the mean; in fact, the same pattern existed for risk perception of other EM wave‐related objects. When an additional multiple linear regression (work not shown in Table) was performed after re‐categorizing objective knowledge into four levels, a higher level of knowledge was still associated with increased risk perception (the highest level; *β*: 0.221, *P*: 0.006) (the third‐highest level; *β*: −0.217, *P*: 0.004) (the fourth‐highest level; *β*: −0.258, *P*: 0.002; reference: the second‐highest level). Furthermore, when multiple linear regression (work not shown in Table) was conducted using the number of questions that the study subjects answered correctly, the risk perception score increased by 0.03 (*P*: 0.003) per one more question that they answered correctly.

On the basis of the above findings, we interpreted that study subjects with greater risk perception have likely been more proactive in gathering relevant information, which led them to perform better on section B. However, the information that study subjects depended on was probably not of high quality as the low mean score on section B (4.6 questions correct out of 13) suggests. Our analysis (work not shown in Table) on sources of information that the study subjects looked up to learn more about EM waves seemed to support our contention; impartial and verified sources of information, such as government or authoritative agencies, were relied on less than exchanges of information with acquaintances. Furthermore, while the study subjects responded as resorting to the internet most frequently to learn more about EM waves, they were not aware of a credible webpage set up by the National Radio Research Agency to improve public knowledge of EMF sources.

The limitation of our study was that its epidemiologic design as a cross‐sectional study limited causal inference. And the study subjects were invited to participate in our survey via e‐mail and KakaoTalk (a widely used messaging application in Korea), which might have resulted in selection bias of over‐representing people who might have been more tolerant to wireless technologies. Furthermore, although section B, which was used to assess the level of objective knowledge in our survey, was more extensive than some other measures used in the past, it might not have functioned as a perfect test of objective knowledge.

However, despite such limitations, several strengths of our study exist. To our knowledge, our study was the first in the literature to investigate factors affecting risk perception of EM waves from 5G network base stations, and our analysis was conducted on a sufficiently large‐sized sample. It is hoped that the results of our study will be reflected in constructing an appropriate risk communication strategy so that reasonable dissemination of technologies can occur.

## Supporting information

Supporting information.
