# Supplementary material for: Factors Affecting Risk Perception of Electromagnetic Waves From 5G Network Base Stations
Source: Bioelectromagnetics. 2020 Aug 31;41(7):491–9. doi: 10.1002/bem.22290 (PMC7540494; doi:10.1002/bem.22290)
Supplement: Supplementary file 1 — Supporting information. [file BEM-41-491-s001.docx]

**Supplementary Table 1. Test of objective knowledge on EM waves (Section B)**

| Question | Answer choice | | |
| --- | --- | --- | --- |
| As more people use mobile phones at the same time, the number of base stations necessary to support connection has to increase | True | False | Do not know |
| As more people use mobile phones at the same time, the level of electromagnetic waves received at base stations would increase | True | False | Do not know |
| Base stations emit same level of electromagnetic waves  throughout the course of the day | True | False | Do not know |
| A specialized protective mobile phone cover exists  that could effectively reduce exposures to electromagnetic waves | True | False | Do not know |
| A specialized metal that deflects electromagnetic waves can be attached  to a mobile phone so that exposures to electromagnetic waves would be reduced | True | False | Do not know |
| Farther you are from base stations, the magnitude of electromagnetic waves  emitted from a mobile phone increases when making a phone call | True | False | Do not know |
| Less electromagnetic waves are emitted from a mobile phone  in the area where there is a better reception | True | False | Do not know |
| When receiving a call closer to base stations, the magnitude of  electromagnetic waves emitted from the stations decreases | True | False | Do not know |
| If you purchase a mobile phone that is designed to emit less electromagnetic waves, you can reduce level of exposures to electromagnetic waves | True | False | Do not know |
| A hands-free mobile phone kit emits higher level of electromagnetic waves  while setting up connection to a mobile phone than making a call | True | False | Do not know |
| If a hands-free mobile phone kit is used, level of  electromagnetic waves absorbed by a brain tissue can be reduced | True | False | Do not know |
| Electromagnetic wave exposure from a mobile phone during a call  is greater when you are farther away from base stations | True | False | Do not know |
| Usually, a mobile phone network with low-power base stations tends to  emit less electromagnetic waves than that with high-power base stations | True | False | Do not know |

**Do not know was counted as incorrect

**Supplementary Table 2. Risk characteristics of EM waves from 5G network base stations**

| Risk characteristic variable | Question | 10-point scale | Mean score  (St. Dev) |
| --- | --- | --- | --- |
| Personal knowledge | How knowledgeable do you believe you are regarding potential health effects due to exposures to EM waves from 5G network base stations? | 1: Not knowledgeable  10: Very knowledgeable | 5.08  (2.30) |
| Controllability | How much control do you believe you have in  preventing potential harms caused by  EM waves from 5G network base stations? | 1: Uncontrollable  10: Very controllable | 4.26  (2.38) |
| Seriousness of risk to the future generations | How serious do you think the effect caused by EM waves from 5G network base station to your children and family members is? | 1: Not serious  10: Very serious | 6.33  (2.05) |
| Dreadfulness | How dreadful do you feel about  EM waves from 5G network base stations? | 1: Not dreadful  10: Very dreadful | 5.77  (2.26) |
| Severity of consequences | How severe do you think the health consequences of exposures to EM waves from 5G network base station are in terms of preventing return to the status quo? | 1: Not severe  10: Very severe | 5.91  (2.16) |
| Risk known to science | How much do you believe that EM waves from 5G network base station are documented by science? | 1: Not known  10: Very well known | 4.84  (2.39) |
| Immediacy of effect of risk | How immediately do you believe that the effect due to exposures to EM waves from 5G network base stations would happen? | 1: Happens slowly  10: Happens fast | 4.60  (2.44) |
| Familiarity | How familiar are you with  EM waves from 5G network base stations? | 1: Unfamiliar  10: Very familiar | 4.64  (2.46) |

**Supplementary Table 3. Comparisons of mean risk perception scores between the two groups divided in respect to level of objective knowledge**

|  | Mean risk perception score (St. Dev) | |  |
| --- | --- | --- | --- |
| EM waves-related object | Low level of  objective knowledge  (n=1,714) | High level of  objective knowledge  (n=1,679) | p-value |
| EM waves from 5G network base stations | 6.63 (1.67) | 7.06 (1.73) | <0.001 |
| EM waves from mobile phones | 6.69 (1.58) | 7.01 (1.66) | <0.001 |
| EM waves from microwaves | 6.86 (1.70) | 7.17 (1.72) | <0.001 |
| EM waves from air fryers | 6.16 (1.72) | 6.43 (1.76) | <0.001 |
| EM waves from hair dryers | 5.98 (1.69) | 6.29 (1.76) | <0.001 |
| EM waves from massage chairs | 6.10 (1.64) | 6.37 (1.71) | <0.001 |
| EM waves from electronic foot baths | 5.71 (1.63) | 5.95 (1.75) | <0.001 |
| EM waves from low frequency therapy devices | 5.84 (1.70) | 6.09 (1.81) | <0.001 |
| EM waves from electric shavers | 5.52 (1.75) | 5.67 (1.83) | 0.016 |
| EM waves from radars | 7.00 (1.81) | 7.42 (1.83) | <0.001 |
| EM waves from transmission lines | 7.78 (1.83) | 8.10 (1.74) | <0.001 |
| EM waves from Bluetooth devices | 6.03 (1.66) | 6.27 (1.75) | <0.001 |
| EM waves from electric heaters | 6.51 (1.65) | 6.69 (1.69) | 0.001 |

**Two-sample T-test was used

**High: study subjects who answered 5-13 questions correctly (above the mean score of 4.6/13)

**Low: study subjects who answered 0-4 questions correctly (below the mean score of 4.6/13)
